# The Hidden Vulnerability: How Underlying SMA Stenosis Turns Routine Intra‐Aortic Balloon Pump Support Into Fatal Mesenteric Ischemia

**DOI:** 10.1155/crcc/4613711

**Published:** 2026-03-13

**Authors:** Nitya Panyala, Bhavneet Singh, Tanya Britto Muthunayagam, Rajeev Mehta

**Affiliations:** ^1^ Internal Medicine, Ball Memorial Hospital, Indiana University School of Medicine, Muncie, Indiana, USA, indiana.edu; ^2^ Pulmonology & Critical Care Medicine, IU Ball Memorial Hospital, Muncie, Indiana, USA

## Abstract

Intra‐aortic balloon pump (IABP) therapy is a widely used mechanical circulatory support device for patients with cardiogenic shock. Although generally safe, IABP insertion can lead to serious complications, including the rare but potentially fatal development of mesenteric ischemia. We report a 58‐year‐old female smoker who presented with ST‐elevation myocardial infarction complicated by cardiogenic shock requiring emergent cardiac catheterization and IABP insertion. Following IABP placement, the patient developed progressive abdominal pain, fever, tachycardia, and bloody bowel movements. CT imaging revealed extensive pneumatosis intestinalis and mural hypoenhancement consistent with small bowel ischemia, along with severe stenosis at the origin of the superior mesenteric artery (SMA). Despite emergent exploratory laparotomy with extensive small bowel resection, the patient developed multiorgan failure and died. This case demonstrates that mesenteric ischemia can occur even when IABP placement appears anatomically appropriate, particularly in patients with underlying visceral vascular disease. The patient′s pre‐existing high‐grade SMA stenosis created a compromised perfusion state where reductions in diastolic blood flow from balloon counterpulsation precipitated catastrophic intestinal ischemia. Clinicians must maintain high clinical suspicion for mesenteric ischemia in patients who develop abdominal symptoms while on IABP support, especially those with visceral atherosclerosis. Early recognition is critical, as delayed diagnosis is almost universally fatal.

## 1. Introduction

Intra‐aortic balloon pump (IABP) therapy is a widely used mechanical circulatory support (MCS) device for patients with cardiogenic shock and high‐risk cardiac interventions. Although generally safe, IABP insertion can lead to serious complications, including the rare but potentially fatal development of mesenteric ischemia.

Patients with pre‐existing visceral vascular disease are at particularly high risk, as baseline compromised perfusion combined with the hemodynamic effects of balloon counterpulsation can precipitate catastrophic intestinal ischemia. We present a case of fatal mesenteric ischemia following IABP insertion in a patient with severe superior mesenteric artery (SMA) stenosis, emphasizing the importance of maintaining clinical suspicion for this devastating complication in vulnerable populations.

## 2. Case Description

A 58‐year‐old female smoker with a history of chronic back pain, gastroesophageal reflux disease, prediabetes, and depression presented to the emergency department with an acute chest pain and dyspnea. Her symptoms began with intermittent shortness of breath the previous day and progressed to severe chest pain around midnight. Emergency medical services administered 324 mg aspirin and DuoNeb with symptomatic improvement.

Upon arrival, a STEMI alert was activated, leading to emergent cardiac catheterization with balloon angioplasty, stent placement in the left anterior descending artery, and IABP insertion for cardiogenic shock. The IABP was inserted percutaneously via the common femoral artery using the Seldinger technique and advanced under fluoroscopic guidance into the descending thoracic aorta, with the proximal balloon tip positioned just distal to the left subclavian artery, confirmed radiographically. The patient was admitted to the intensive care unit where she developed hypoxic respiratory failure requiring intubation and mechanical ventilation. She initially demonstrated hemodynamic improvement and was maintained on a low‐dose nitroglycerin infusion for blood pressure control.

On the following hospital day, the patient′s clinical status deteriorated. Starting in the afternoon at approximately 1–2:00 PM, she developed worsening abdominal pain accompanied by fever, tachycardia, hypotension, and bloody bowel movements. The nitroglycerin infusion was discontinued due to hypotension.

**Figure 1:**
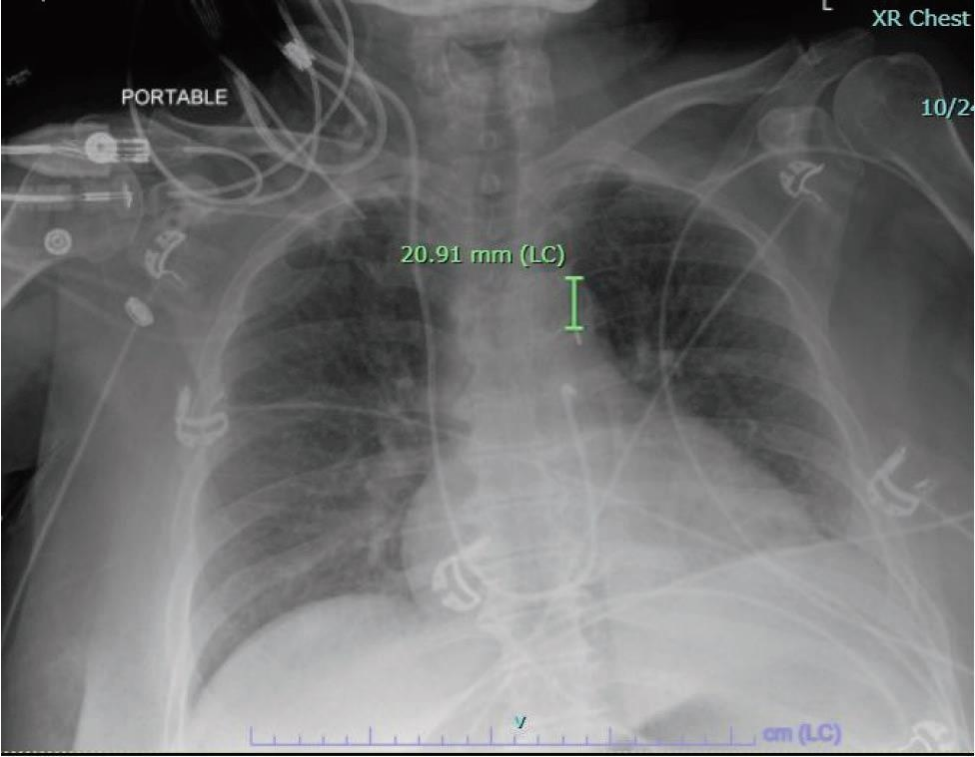
Radio‐opaque marker approximately 2 cm distal from the aortic knob.

The balloon pump was initially weaned to 3:1 but returned to 1:1 support with heparin discontinuation (ACT < 170). Multidisciplinary consultations with general surgery and gastroenterology were obtained after KUB imaging suggested a possible small bowel obstruction. CXR was done which confirmed correct placement of the balloon pump (Figure 1). The patient was made NPO and continued on IV proton pump inhibitor therapy with anticoagulation modified to aspirin and ticagrelor per cardiology recommendations.

**Figure 2:**
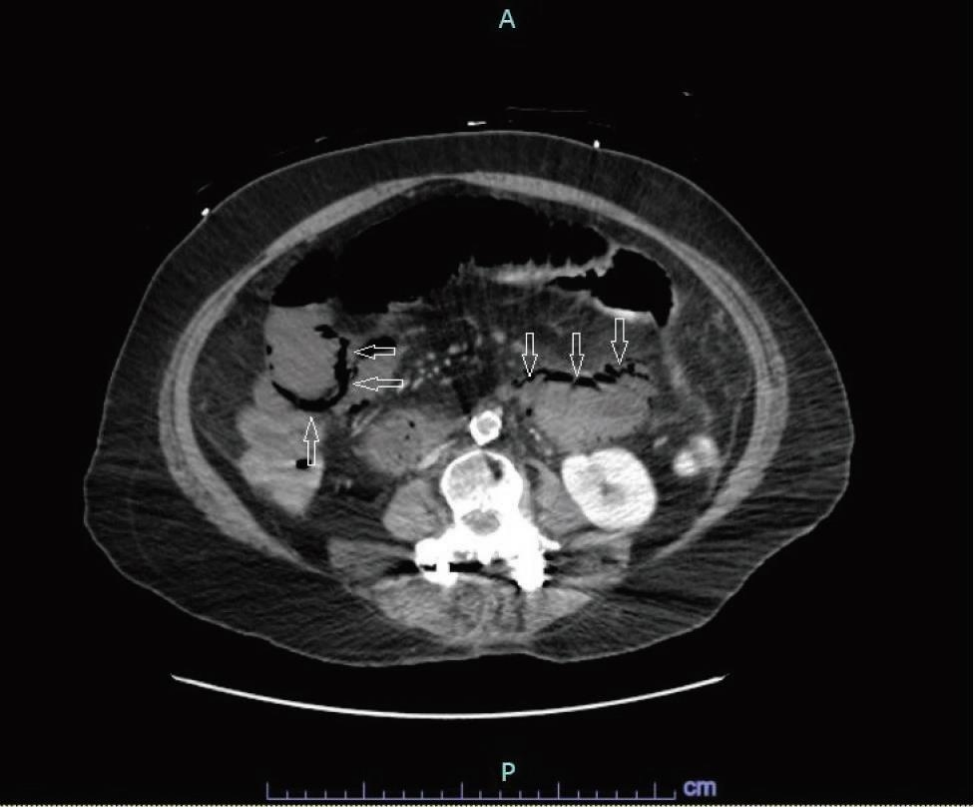
Small bowel ischemia with extensive pneumatosis intestinalis.

**Figure 3:**
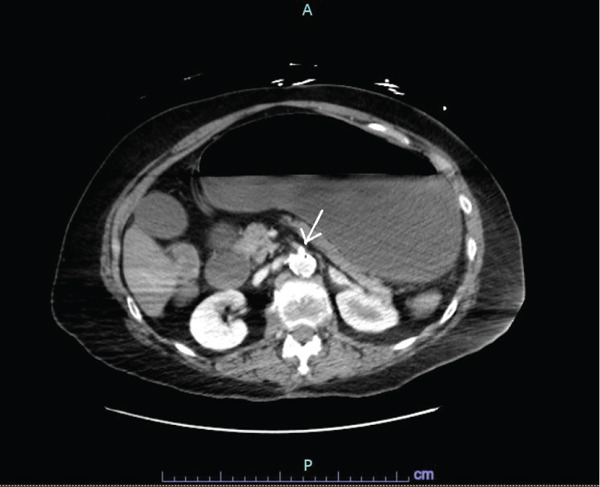
Severe stenosis at origin of superior mesenteric artery (SMA).

A stat CT scan of the abdomen and pelvis with intravenous contrast was obtained approximately 2 h after the start of abdominal symptoms, revealing findings highly concerning for small bowel ischemia, including extensive pneumatosis intestinalis (Figure 2) and mural hypoenhancement of the proximal to mid‐small bowel. Associated small bowel dilation was likely representative of adynamic ileus rather than mechanical obstruction. Imaging also demonstrated severe SMA origin (Figure 3) and left common iliac artery stenosis, with suspected chronic occlusion or high‐grade stenosis of the celiac artery.

The patient was taken emergently to the operating room at approximately 6:30 PM for exploratory laparotomy, which revealed extensive small bowel ischemia requiring resection, leaving approximately 75 cm of viable bowel. Postoperatively, she developed progressive multiorgan failure, manifested by severe lactic acidosis (lactate 8.3 mmol/L), marked transaminitis (ALT/AST 635/633 U/L), and acute kidney injury with a creatinine of 1.66 mg/dL from a normal baseline the prior day. In the setting of this multiorgan failure, she developed hemodynamic instability requiring initiation of low‐dose norepinephrine infusion (4–5 *μ*g/min), having not required vasopressor support preoperatively. Following discussions with the family that were consistent with the patient′s previously expressed goals of care, comfort‐focused measures were initiated.

## 3. Discussion

Mesenteric ischemia is a rare but potentially fatal complication of IABP therapy, often underdiagnosed due to its nonspecific early presentation. Although IABP counterpulsation is widely used for hemodynamic support, vascular complications remain a concern. A comprehensive review of 20 studies including > 23,000 patients reported an overall vascular complication rate of 0.94%–31.1%, reflecting heterogeneity in populations, device sizes, and definitions. Limb ischemia was most common, whereas mesenteric ischemia was infrequently reported. Only a small number of studies have specifically assessed mesenteric ischemia in IABP‐treated patients, and the reported clinical incidence appears to be low. In most series, mesenteric ischemia is rare; for example, one study reported a single case (0.9%) that ultimately resulted in patient death, whereas another demonstrated compromise of SMA flow in 61 of a subset of 63 patients (87%), based on imaging assessment, suggesting that subclinical or radiographically detectable mesenteric hypoperfusion may be far more common than clinically recognized ischemia [1].

This case highlights that mesenteric ischemia can occur even when IABP placement appears appropriate on imaging, particularly in the presence of significant underlying visceral vascular disease. During diastole, balloon inflation augments diastolic pressure and enhances coronary perfusion; however, in patients with compromised splanchnic circulation, this augmentation may paradoxically impair mesenteric blood flow [2]. In this patient, high‐grade stenosis at the origin of the SMA likely created a low‐perfusion state in which even modest IABP‐related reductions in diastolic flow were sufficient to precipitate intestinal ischemia.

Supporting this mechanism, direct hemodynamic studies in a porcine model using surgically placed perivascular flow probes demonstrated that IABP counterpulsation is associated with significant reductions in SMA flow during early and mid‐diastole, with incomplete recovery later in diastole and overall systolic SMA flow remaining lower than baseline [3]. These findings provide physiologic evidence that IABP inflation–deflation cycles can induce phase‐specific splanchnic hypoperfusion, particularly in patients with limited mesenteric perfusion reserve.

Mesenteric ischemia may be classified as occlusive or nonocclusive. In some cases, emboli originating from an atherosclerotic aorta can obstruct mesenteric vessels. Nonocclusive mesenteric ischemia typically results from intense vasoconstriction of the SMA in the setting of reduced splanchnic perfusion, often due to hypotension or vasopressor use [4]. In this case, pre‐existing high‐grade stenosis at the origin of the SMA significantly impaired mesenteric perfusion, rendering the bowel highly vulnerable to any transient or sustained reductions in blood flow.

Several mechanisms have been implicated in IABP‐associated visceral ischemia. One study identified malpositioning due to inappropriate balloon length and proximal tip placement as a key contributor, leading to unintended compromise of visceral arterial branches despite radiographically correct positioning [5]. This case demonstrates that anatomically correct IABP placement does not eliminate the risk of mesenteric ischemia, particularly in patients with underlying vascular stenosis. In such individuals, baseline mesenteric perfusion is already compromised, leaving little reserve to tolerate further reductions in diastolic flow induced by balloon counter pulsation.

CT imaging in our patient revealed extensive pneumatosis intestinalis and mural hypoenhancement, consistent with advanced bowel infarction. Pneumatosis—gas within the bowel wall—is a well‐recognized radiologic marker of transmural necrosis, typically resulting from mucosal disruption and bacterial translocation [6].

A similar case was reported in which a woman developed a fatal pneumatosis intestinalis while on IABP support. The authors emphasized that patients with small‐caliber aortas and significant atherosclerotic disease are at heightened risk of visceral ischemia, even when balloon size and positioning conform to current guidelines [7].

Beyond IABP support, mesenteric ischemia has also been reported, albeit infrequently, across other MCS modalities. In Impella‐supported patients, the literature primarily describes acute limb ischemia, particularly in those with pre‐existing peripheral arterial disease or small‐caliber femoral arteries, whereas reports of mesenteric ischemia remain scarce [8]. It is reasonable to hypothesize that Impella‐supported patients could develop splanchnic hypoperfusion if reduced pulsatility, device malposition, or inadequate unloading impair systemic blood flow, especially in severe cardiogenic shock [9].

Intestinal ischemia has also been described in patients receiving extracorporeal membrane oxygenation (ECMO), though reports remain limited. Colonic ischemia has been reported during venovenous extracorporeal membrane oxygenation (VV‐ECMO), where hemodynamic derangements and thrombotic complications are recognized contributors, though the precise mechanisms remain incompletely understood [10]. In VA‐ECMO, abnormal flow dynamics such as retrograde aortic flow, large zones of aortic stasis, insufficient left‐ventricular unloading, and watershed effects may impair end‐organ perfusion despite seemingly stable systemic hemodynamics [11].

Although the underlying mechanisms differ across MCS devices, intestinal ischemia is consistently multifactorial and often recognized late due to sedation and nonspecific symptoms. As the use of Impella, VA‐ECMO, and the IABP continues to expand, careful patient selection and heightened vigilance for splanchnic hypoperfusion remain essential.

Clinicians must maintain a high index of suspicion for mesenteric ischemia in patients who develop abdominal symptoms while on IABP and other MCS support devices. In patients with known or suspected visceral atherosclerosis, early vascular imaging may influence the choice of mechanical support modality. Early recognition remains critical, as delayed diagnosis of mesenteric ischemia is almost universally fatal.

## Funding

No funding was received for this manuscript.

## Conflicts of Interest

The authors declare no conflicts of interest.

## Data Availability

Data sharing is not applicable to this article as no datasets were generated or analyzed during the current study.
